# Tauroursodeoxycholic acid (TUDCA) alleviates endoplasmic reticulum stress of nuclear donor cells under serum starvation

**DOI:** 10.1371/journal.pone.0196785

**Published:** 2018-05-02

**Authors:** Ying Zhang, Pengxiang Qu, Xiaonan Ma, Fang Qiao, Yefei Ma, Suzhu Qing, Yong Zhang, Yongsheng Wang, Wei Cui

**Affiliations:** 1 Key Laboratory of Animal Biotechnology of the Ministry of Agriculture, College of Veterinary Medicine, Northwest A&F University, Yangling, Shaanxi, PR China; 2 Engineering Center for Animal Embryo Technology, Yangling, Shaanxi, PR China; 3 Laboratory of Embryo Technology in Livestock, Northwest A&F University, Yangling, Shaanxi, PR China; 4 Department of Gynecology and Obstetrics, Tangdu Hospital, the Fourth Military Medical University, Xi'an, Shannxi Province, PR China; 5 Department of Veterinary and Animal Sciences, University of Massachusetts, Amherst, MA, United States of America; 6 Animal Models Core Facility, Institute for Applied Life Sciences (IALS), University of Massachusetts, Amherst, MA, United States of America; Peking University Third Hospital, CHINA

## Abstract

Serum starvation is a routine protocol for synchronizing nuclear donor cells to G0/G1 phase during somatic cell nuclear transfer (SCNT). However, abrupt serum deprivation can cause serious stress to the cells cultured *in vitro*, which might result in endoplasmic reticulum (ER) stress, chromosome damage, and finally reduce the success rate of SCNT. In the present study, the effects of tauroursodeoxycholic acid (TUDCA), an effective ER stress-relieving drug, on the nuclear donor cells under serum deprivation condition as well as following SCNT procedures were first assessed in the bovine. The results showed that TUDCA significantly reduced ER stress and cell apoptosis in those nuclear donor cells. Moreover, it significantly decreased the expression of *Hdac1* and *Dnmt1*, and increased the level of H3K9 acetylation in nuclear donor cells compared with control group. SCNT reconstructed embryos cloned from TUDCA-treated donor cells showed significantly higher fusion, cleavage, blastocyst formation rate, total cell number in day 7 blastocysts, and lower apoptotic index than that from control group. In addition, the expression of *Hdac1*, *Dnmt1* and *Bax* was significantly lower in blastocysts derived from TUDCA-treated donor cells than that from control group. In conclusion, TUDCA significantly reduced the ER stress of nuclear donor cells under serum starvation condition, and significantly improved the developmental competence of following SCNT reconstructed embryos when these TUDCA-treated cells were used as the nuclear donors.

## Introduction

Though somatic cell nuclear transfer (SCNT) has been successful in mammalian species for more than two decades [1], the efficiency of this technique is still very low, which hampers its wide use [2–4]. Numerous works have been done to improve the efficiency of SCNT, and great achievement has been obtained since the birth of “Dolly”. The cloning efficiency has increased from 1–2% to about 10% in most mammalian species. However, the success rate is still significantly lower than their fertilized counterparts. In addition, most cloned offspring suffer from various abnormalities, such as large offspring symptom, large umbilical veins and arteries, pulmonary atelectasis, limb contracture, and respiratory disorders[5–7]. Therefore, endeavor is still urgently needed to improve the efficiency of this promising technique.

As a technique with complex procedures, multiple factors might influence the final efficiency of SCNT, including the quality of recipient oocytes, tissue origin of nuclear donor cells, activation protocols, and the culture conditions. The coordination of cell cycle phases between donor nuclei and recipient cytoplasm is essential to maintain correct ploidy and reduce DNA damage in SCNT embryos [8]. Numerous studies indicated that donor cells at G0/G1 phase are more conducive to the following development of SCNT embryos [9–11]. Serum starvation is a commonly used protocol to induce nuclear donor cells arrested at G0/G1 phase, and has been regarded as an important step for the success of SCNT [12, 13]. However, cells cultured *in vitro* are susceptible to the culture environment, and abrupt serum deprivation can cause serious stress to these cells, which may result in cellular damages in various aspects, including the metabolic disorders, instability of membrane and chromosome, and increasing oxygen free radicals in cytoplasm. All these damages within nuclear donor cells might finally influence the success of SCNT [14–16]. Studies also confirmed that improved donor cell quality is beneficial to the developmental competence of reconstructed embryos. For example, treatment of donor somatic cells with apoptosis inhibitor hemoglobin and beta-mercaptoethanol [17] or with antioxidant melatonin[18] can significantly improve the developmental competence of cloned embryos in bovine and porcine, respectively.

In eukaryotes, endoplasmic reticulum (ER) stress is a protective stress reaction mediated by unfolded protein response (UPR) under normal physiological or mild adverse condition [19, 20]. UPR sensors are highly regulated by the formation of dynamic protein scaffolds, leading to changes of cellular epigenetic, which make the cells adapted to the adverse condition [21, 22]. However, if stress is too severe or lasts too long, excessive unfolded or misfolded proteins will be accumulated in the cytoplasm, which may then disturb normal physiological activities and finally lead to damages of cellular organelles and nucleus [23]. In SCNT procedure, serum starvation is a common protocol to synchronize nuclear donor cells at G0/G1 phase. However, this SCNT-required procedure is also an obvious stress for cultured cells, which might result in serious ER stress. Previous studies have shown that tauroursodeoxycholic acid (TUDCA), an effective inhibitor of ER stress, can significantly relieve ER stress and block apoptosis [24, 25]. Its beneficial effects on oocyte maturation in porcine [26] and preimplantation embryo development in both porcine [27–29] and bovine [30] are also recently confirmed by different laboratories. However, whether TUDCA-treatment might alleviate the ER stress of nuclear donor cells under serum starvation and hence increase the developmental potency of bovine SCNT embryos using these treated donors has not been studied or reported.

In the present study, the effects of TUDCA on ER stress relief of nuclear donor cells under serum starvation condition were assessed in terms of apoptotic index, cell cycle status, histone acetylation, and the expression of several ER and development-related genes in cow. In addition, its effects on the development of reconstructed embryos after SCNT were also evaluated.

## Materials and methods

This study was carried out in accordance with the guidelines for the care and use of animals of Northwest A&F University. All animal experimental procedures were approved by Animal Care Commission of College of Veterinary Medicine, Northwest A&F University. Except as otherwise noted, all chemicals were purchased from Sigma-Aldrich (St. Louis, USA).

### Establishment of the nuclear donor cell

A 40-day-old bovine fetus (Holstein breed) was collected from a local slaughterhouse (Tumen abattoir, Xi’an, Shaanxi, China) and transported to the laboratory in 0.9% sterile saline containing 100 IU/mL penicillin and 100 mg/L streptomycin sulfate within 3–4 h at 15–20°C. Fetal skin tissue was dissected and then washed three times in PBS, afterward was cut into about 1 mm^2^ per pieces, and rinsed twice in Dulbecco modified Eagle medium (DMEM). The tissue pieces were placed in a 60-mm dish with DMEM containing 10% fetal bovine serum (FBS) in 5% CO_2_ at 39°C.

### Cell cycle and apoptosis analysis

The effects of serum starvation and TUDCA-treatment on the cell cycle and apoptosis of nuclear donor cells were examined according to the experimental designs. Cells were digested by 0.25% trypsin in D-Hanks solution for 3 min at 37°C, rinsed 2 times with chilled PBS, fixed in 75% ethanol by freezing for 1 h at -20°C, rinsed once with chilled PBS again, resuspended with 400 μL cold PBS containing 20 μL of RNase A, incubated at 37°C for 30 min, filtered through a 400-mm mesh, mixed with 400 μL of propidium iodide (100 mg/mL) staining solution in the dark, incubated for 1 h at 4°C, and finally examined by flow cytometry (Becton-Dickinson, Oxford, UK). For cell apoptosis analysis, cells were collected by trypsin digestion. After washing 3 times with cold PBS, 400 μL of annexin V (BestBio, BB-4101) and 5 μL of annexin V-EGFP staining medium (BestBio, BB-4101) were added. The cell mixture was mixed slightly, incubated in the dark for 15 min at 4°C, supplemented with 10 μL of propidium iodide (10 mg/mL), incubated for 5 min, and finally examined by flow cytometry (Becton-Dickinson).

### H3K9ac immunofluorescence

Immunofluorescence was processed as previously described [16, 31]. Cells were washed 3 times in PBS, fixed at room temperature for 30 min, washed again, permeabilized with 0.2% Triton X-100 for 30 min, blocked at 4°C for 12 h, washed 3 times by PBS, then incubated with primary antibody of anti-H3K9ac (ab10812, Abcam, Cambridge, UK) at 4°C for 12 h, washed 3 times by PBS, incubated with Alexa Fluor 488-labeled secondary antibody in the dark at room temperature for 2 h, and washed 3 times by PBS. Nuclear labeling was performed with 4,6-diamidino-2-phenylindole hydrochloride (DAPI, Vysis Inc., Downers Grove, USA) for 3 min, and washed 3 times in 0.1% PBS-PVA for 5 min each. Finally, samples were inspected by epifluorescence using a Nikon eclipse Ti-S microscope (Nikon, Tokyo, Japan). All images were captured using Nikon DS-Ri1 digital camera under same parameters. The levels of histone acetylation were quantified by the fluorescence intensity using Image-Pro plus 6.0 software (Media Cybernetics, Silver Spring, USA) with DAPI channel as the normalizer. The experiment was replicated 3 times, with around 15 samples per group in each replication.

### Production of SCNT embryos

SCNT embryos were produced as illustrated before [16, 32]. Briefly, Holstein cow ovaries were collected from the same local abattoir as mentioned above, and transported to the laboratory in 0.9% sterile saline within 3–4 h at 15–20°C. Ovaries were washed several times in pre-warmed 0.9% sterile saline. The follicle liquid and cumulus–oocyte complexes (COCs) were retrieved from antral follicles (2-8mm in diameter) with 10 mL syringe. Under stereomicroscope, COCs with at least three intact layers of cumulus cells and homogenous cytoplasm were collected in PBS supplemented with 5% (v/v) FBS. After three washing in oocyte maturation medium (TCM-199 supplemented with 10% FBS, 1μg/mL 17 β-estradiol, 0.075 IU/mL human menopausal gonadotropin, and 30 ng/mL epidermal growth factor), about 100 COCs were placed in a 35 mm petri-dish containing 4 mL oocyte maturation medium, and incubated at 38.5ºC in a humidified incubator of 5% CO_2_ in air for 22 h. After maturation, cumulus cells were removed by treatment with 0.2% bovine testicular hyaluronidase in PBS. Oocytes with a polar body were selected for SCNT. Oocytes were enucleated by aspirating the first polar body and a small amount of surrounding cytoplasm using a beveled glass pipette with 20 μm internal diameter in PBS supplemented with 7.5 μg/mL cytochalasin B and 10% FBS. Successfully enucleated oocytes were selected under a fluorescence microscopy after staining with Hochest 33342. A donor cell was injected into the perivitelline space of the successfully enucleated oocytes, and then fused using two closely spaced electrical pulses of 35 V for 10 μs. The reconstructed oocytes were kept in modified synthetic oviduct fluid (mSOF) containing 5 μg/mL cytochalasin B for 2 h before activation. The reconstructed oocytes were then activated in 5 μM ionomycin for 4 min followed by 4 h exposure to 1.9 mM 6-dimethylaminopurine. Reconstructed embryos were washed three times in mSOFaa, and finally cultured in 200 μL droplets (40 embryos per droplet) of mSOFaa. The culture condition was humidified atmosphere of 5% CO_2_ in air and 38.5°C. Development to two-cell embryos and blastocysts were monitored at 24 h and 168 h of culture, respectively (0 h being the time that embryos were transferred to mSOFaa).

### Quantitative real-time PCR (qPCR)

Method of qPCR for donor somatic cells: total RNA was extracted from cells using Qiazol (Qiagen, Germany) according to the manufacturer’s protocol. The reverse transcription (RT) reaction was performed using the M-MLV reverse transcriptase according to the manufacturer’s protocol (Ambion Co., USA). The cDNA products were immediately used for real-time PCR. Method of qPCR for embryos: thirty embryos of each group were used for qPCR, the reactions were carried out in triplicate with the same pool of embryos, and experiments were replicated three times. Embryos were lysed using the lysis buffer of Cells-to-Signal™ Kit (Ambion Co., USA) according to the manufacturer's protocol. The reverse transcription (RT) reaction was performed using M-MLV Reverse Transcriptase included in the kit. All qPCR reactions were performed on the quantitative real-time PCR StepOne plus system (ABI, Carlsbad, CA, USA), including ROX 0.4 μL, SYBR Green I 10 μL, F-primer 0.8 μL, R-primer 0.8 μL, embryos cDNA 2 μL and dH_2_O 6 μL, with steps: 95ºC 30 sec, followed by 40 cycles of 95ºC 5 sec, 60ºC 30 sec and 72ºC 30 sec. Two housekeeping genes, beta-actin and histone 2a.2 (H2A.2), were used as reference genes. Primer sequences are shown in Table 1.

**Table 1:** Primers for q-PCR.

Gene	Sequence	Product Size (bp)	Tm(°C)	Gene bank accession No.
*β-actin*	F (5'- 3') AAGGACCTCTACG CCAACACG	255	60	AY141970
	R (5'- 3') GAAGCATTTGCGG TGGACGAT			
*H2A*.*2*	F (5'- 3') GAGGAGCTGAACAAGCTGTTG	144	60	BF076713
	R (5'- 3') TTGTGGTGGCTCTCAGTCTTC			
*Bip*	F (5'- 3') GCTATTGCTTATGGCCTGGA	167	60	NM_001075148.1
	R (5'- 3') CGCTGGTCAAAGTCTTCTCC			
*Chop*	F (5'-3') GTCACTGCCTTTCTCCTTCG	218	60	NM_001078163.1
	R (5'- 3') GGGAGGTGTGTGTGACCTCT			
*Ire1*	F (5'- 3') GCTATTGCTTATGGCCTGGA	170	60	XM_001789477.1
	R (5'- 3') CTCCATGGCGATCATCTTCT			
*Bax*	F (5'- 3') TTTGCTTCAGGGTTTCATCC	246	60	NM_173894.1
	R (5'- 3') CAGTTGAAGTTGCCGTCAGA			
*Bcl-2*	F (5'- 3') ATGTGTGTGGAGAGCGTCAA	137	60	NM_001166486.1
	R (5'- 3') TACAGCTCCACAAAGGCGTC			
*Hdac1*	F (5'-3') ATCGGTTAGGTTGCTTCAATCTG	168	60	BT030718.1
	R (5'- 3') GTTGTATGGAAGCTCATTAGGGA			
*Dnmt1*	F (5'- 3') CTCAGAAGGGAGATGTGGAG	138	60	NM_182651.2
	R (5'- 3') AGTAGTCACAGTAGCTGAGGA			

F: forward primer; R: reverse primer.

### Detection of apoptosis and total cell number in blastocysts

The apoptotic index of day 7 blastocysts was examined by DeadEnd Fluorometric TUNEL System (Promega, Madison, WI, USA) according to the manufacturer’s protocol. Blastocysts were fixed in 4% paraformaldehyde for 2 h at room temperature, and permeabilized in 0.5% Triton X-100 for 5 min. After being balanced in buffer for 5–8 min, they were incubated with FITC-conjugated dUTP (5 μL), terminal deoxynucleotidyl transferase (1 μL) and buffer (45 μL) at 37ºC for 1 h in the dark (hereafter, all manipulations were performed in the dark). The tailing reaction was terminated in 2×SSC (SSC: 0.15 M sodium chloride, 0.015 M sodium citrate) for 15 min. Then the embryos were incubated in PBS containing 25 μg/mL RNase A and DAPI for 3 min, washed three times with PBS-PVA for 5 min, and mounted on slides for observation under a Nikon Eclipse Ti-S microscope (Nikon, Tokyo, Japan). All images were captured using Nikon DS-Ri1 digital camera and the nuclei were identified by their blue fluorescence, and nuclear DNA fragmentation was labeled with FITC appeared green. For total cells counting, embryos were fixed with 4% (v/v) paraformaldehyde and then washed in PBS-PVA. After being labeled with DAPI and washed in PBS-PVA, blastocysts were mounted, and images were captured using same microscope and digital camera mentioned above.

### Experimental design

In experiment 1, the optimum TUDCA concentration was determined. Fibroblasts at third passage were cultured in DMEM containing 10% FBS (normal condition) or 0.5% FBS (serum starvation condition) with TUDCA at different concentrations (0, 50, 100, 150 or 200 μM) for 48 h. Thereafter, the expression of several ER stress marker genes, including *Ire1*, *Bip*, and *Chop*, were examined to evaluate the effects of different TUDCA concentrations on ER stress of bovine fibroblasts.

In experiment 2, effects of TUDCA treatment on the nuclear donor cells were further assessed in terms of cellular cycle stage, apoptotic index, expression of several reprogramming related genes, and H3K9 acetylation level.

In experiment 3, the effects of TUDCA-treated nuclear donor cells on the development of SCNT reconstructed embryos were compared with control group. The quality of embryos was assessed in terms of cleavage rate, blastocyst formation rate, total cell number, apoptotic index and gene expression of day 7 blastocysts.

### Statistical analysis

Experiments were repeated at least thrice, and each replicate was performed using oocytes matured on the same day to remove any batch variations. All embryos were allocated randomly to each treatment group. Fusion rate, cleavage rate and blastocyst formation rate were analyzed with χ^2^ test. The total cell number and apoptotic index were analyzed using one-way ANOVA. The relative abundance of gene transcripts was determined by testing the data for normality and equal variance using the Levene median test, ANOVA, and followed multiple pair wise comparisons using the Tukey’s test. Statistical analyses were conducted using the SPSS software package (SPSS Inc., Chicago, IL, USA). Data were expressed as mean ± SEM. A p value less than 0.05 was considered statistically significant.

## Results

### TUDCA relieves ER stress of nuclear donor cells under serum starvation

**Fig 1:**
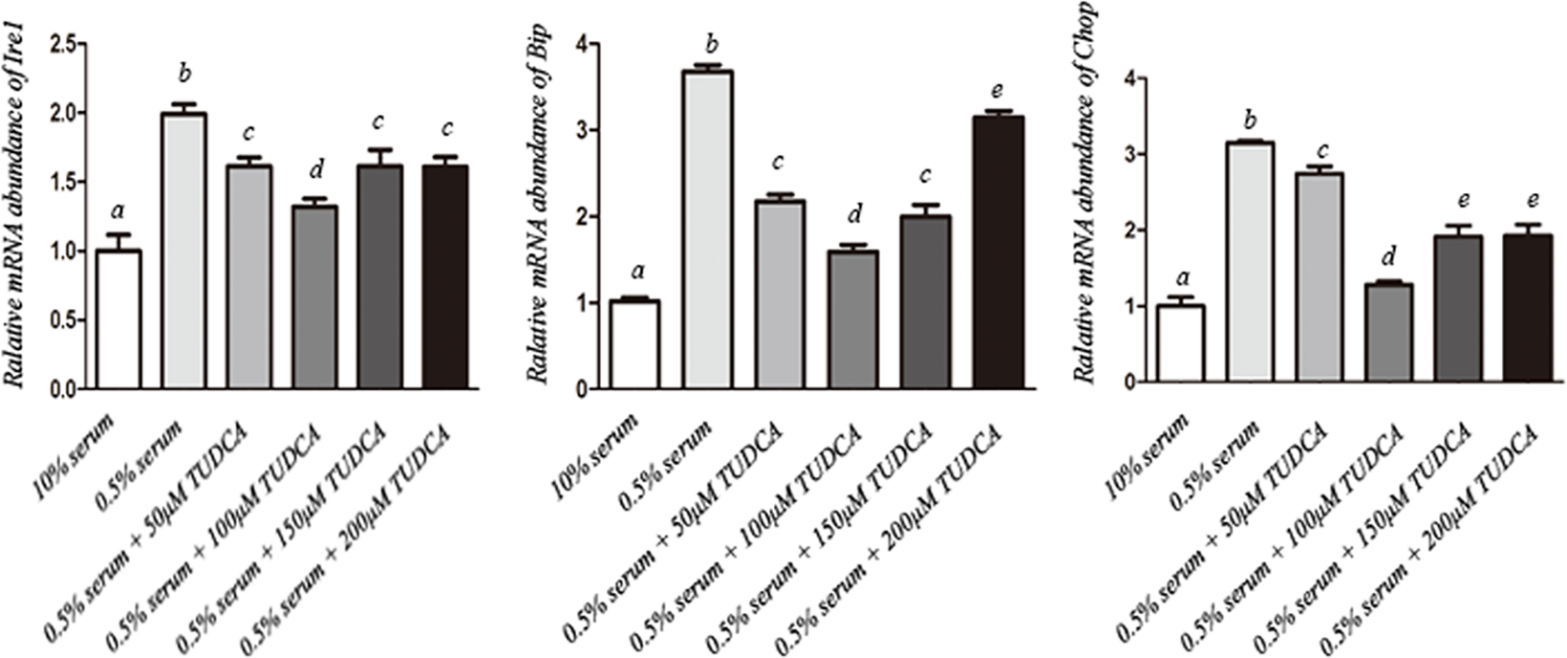
Relative expression levels of endoplasmic reticulum (ER) stress related genes in donor fibroblast cells cultured in medium containing 10% FBS (normal condition) or 0.5% FBS (serum starvation condition) with TUDCA at different concentrations (0, 50, 100, 150 and 200 μM). a-e: different letters indicate significant difference (P<0.05).

As shown in Fig 1, serum starvation caused significantly higher expression of *Ire1*, *Bip* and *Chop* in fibroblasts when compared with that from 10% serum containing group (P<0.05), indicating that serum deprivation might result in serious ER stress. Supplementation of TUDCA significantly reduced the expression of all these ER stress related genes in a concentration-dependent manner, with lowest level at 100 μM. These data demonstrated that TUDCA could reduce the ER stress of nuclear donor cells caused by serum deprivation, and 100 μM TUDCA was the optimal concentration for somatic cell treatment under our current conditions.

### Effects of TUDCA on functional properties of nuclear donor cells

**Fig 2:**
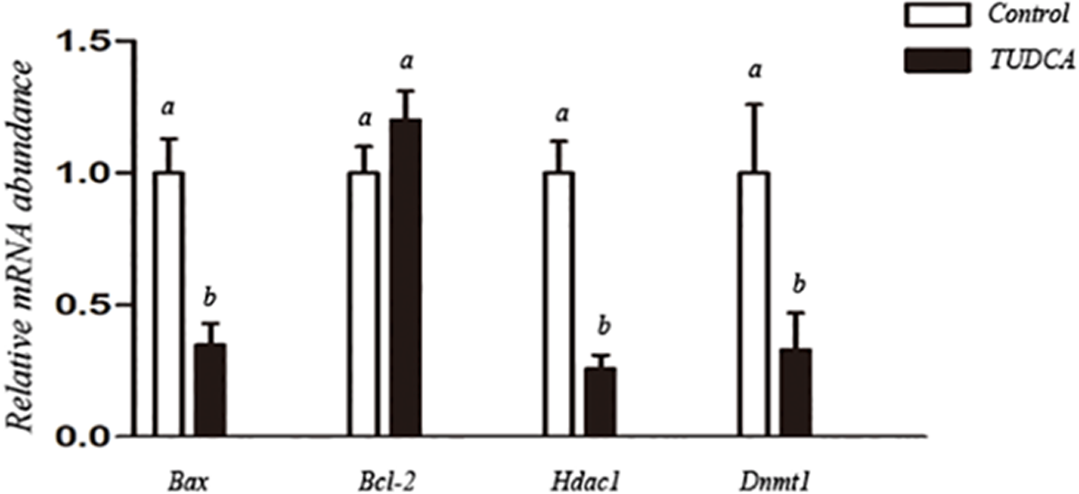
Relative abundance of apoptosis-related genes (anti-apoptosis marker *Bcl-2*, pro-apoptosis marker *Bax*) and reprogramming-related genes (*Hdac1* and *Dnmt1*) in control and TUDCA-treated donor cells. a,b: different letters indicate significant difference (P<0.05).

**Fig 3:**
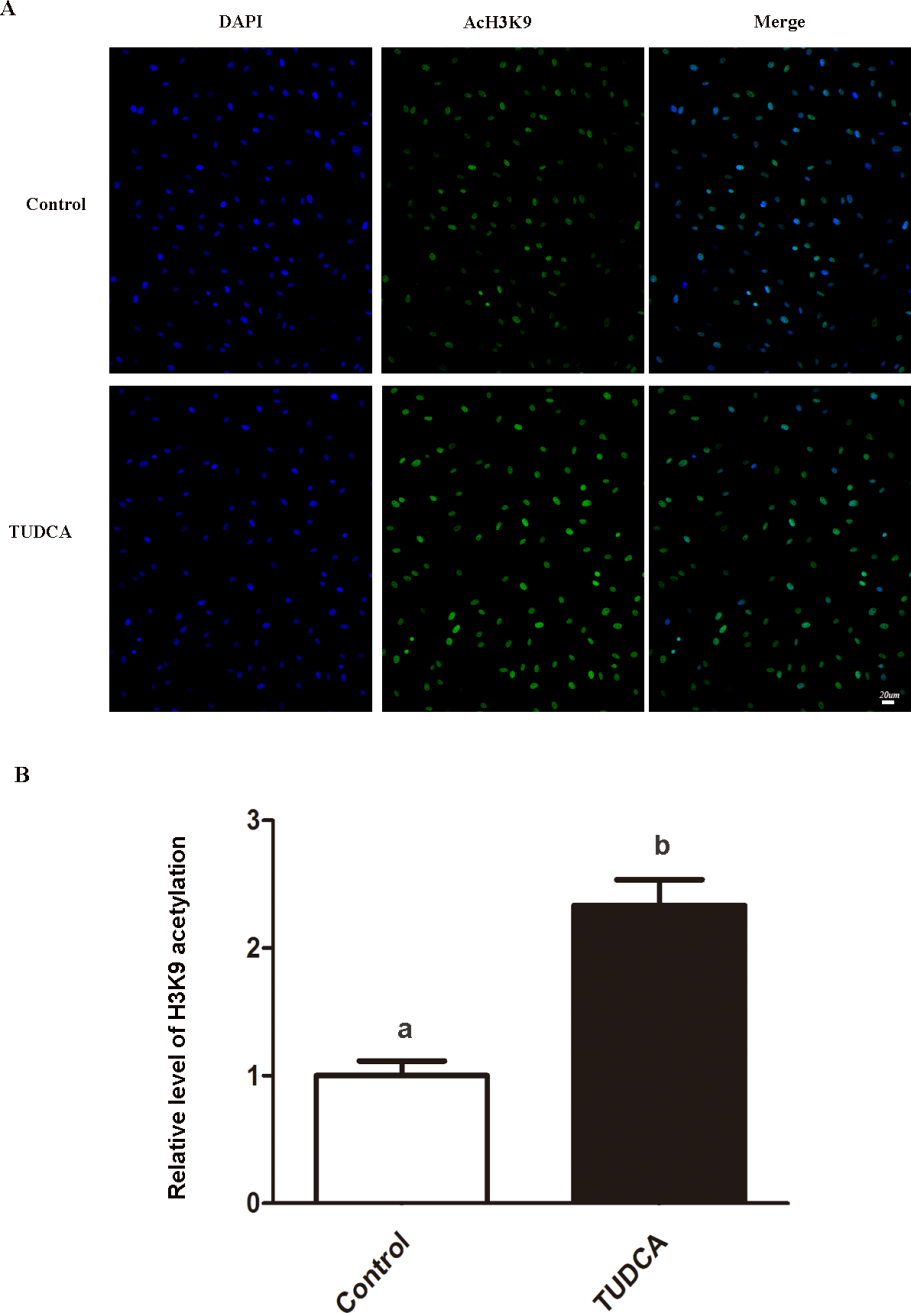
Global level of H3K9 acetylation in control and TUDCA-treated donor cells. (A) H3K9 acetylation was stained as green, nuclei were counterstained with DAPI to visualize as blue. Scale bar: 20 μm. (B) Quantification of H3K9 acetylation/DNA signal intensities. a,b: different letters indicate significant difference (P<0.05).

To further evaluate effects of TUDCA on functional properties of nuclear donor cells under routine serum starvation condition, experiments were performed between control group (no TUDCA addition) and TUDCA group (100 μM TUDCA supplementation). Though there was no significant difference regarding expression level of anti-apoptosis marker *Bcl-2*, the expression level of pro-apoptosis marker *Bax*, and epigenetic modification related genes *Hdac1* and *Dnmt1*, were significantly lower in TUDCA treatment group than control group (Fig 2). These results suggested that TUDCA treatment might alleviate cell apoptosis and regulate cellular growth and epigenetic modification. Therefore, we further investigated its effects on cellular status and epigenetic modification by flow cytometry and H3K9 acetylation immunofluorescence staining, respectively. As shown in Table 2, TUDCA did not affect cell cycle status under routine serum starvation, both groups had more than 95% fibroblasts in G0/G1 phase and very low percentages of fibroblasts in S and G2/M phases. Compared with control group, TUDCA treatment significantly decreased the percentage of apoptotic cells (Table 3). Immunofluorescence staining showed that level of H3K9 acetylation was 2.3-fold higher in TUDCA treatment group than that in the control group (Fig 3).

**Table 2:** Percentages of fibroblasts at different cell cycle phases under control and TUDCA treatment conditions.

Group	Percentages of fibroblasts at different cell cycle phases (%)		
	G0/G1	S	G2/M
Control	95.65±2.91	2.37±1.21	1.98±1.76
TUDCA	95.95±2.65	1.58±1.54	2.47±1.64

No significant difference between Control and TUDCA treatment groups (P>0.05).

**Table 3:** Apoptosis analysis of donor cells in control and TUDCA treatment groups.

Group	Annexin-negative	Apoptosis	Necrosis	Late apoptosis
Control	90.2±1.3^a^	5.4±0.4^a^	1.0±0.3	3.4±1.0
TUDCA	95.9±0.9^b^	1.1±0.2^b^	1.5±0.5	1.5±0.7

Apoptosis was detected by annexin V/propidium iodide (PI) staining assay. Annexin-negative: cells without any annexin or PI signal; Apoptosis: cells with annexin signal only; Necrosis: cells with PI signal only; Late apoptosis/necrosis: cells with annexin and PI signals.

a,b: different superscripts within same column indicate significant difference (P<0.05).

### TUDCA-treatment on nuclear donor cells improves following development of SCNT reconstructed embryos

**Fig 4:**
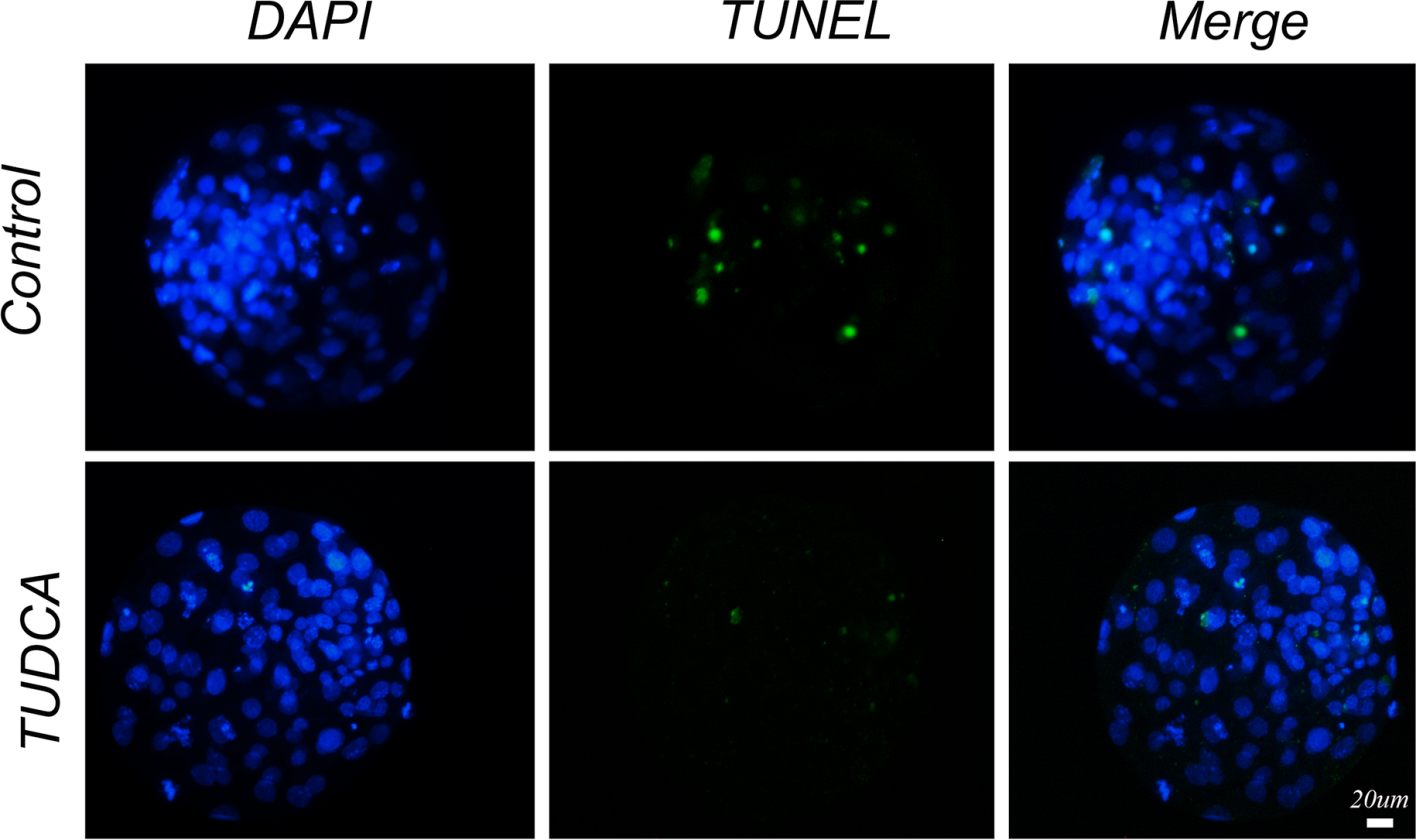
The apoptotic index in SCNT reconstructed blastocysts derived from control or TUDCA-treated donor cells. Apoptotic nuclei were stained as green by TUNEL assay, DNA was counterstained with DAPI to visualize as blue. Scale bar: 20 μm.

**Fig 5:**
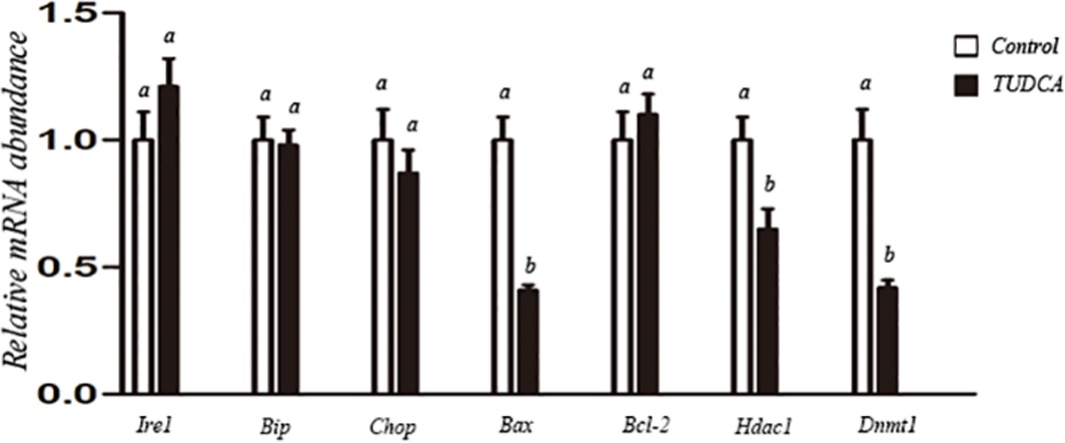
Relative mRNA abundance of *Ire1*, *Bip*, *Chop*, *Bax*, *Bcl-2*, *Hdac1* and *Dnmt1* genes in SCNT reconstructed blastocysts derived from control or TUDCA-treated donor cells. a,b: different letters indicate significant difference (P<0.05).

As shown in Table 4, nuclear donor cells derived from TUDCA-treatment group showed significantly higher fusion rate than control group (85.8% vs 68.9%, P<0.05). Moreover, the cleavage rate (93.2% vs 86.6%, P<0.05) and SCNT blastocyst formation rate (37.9% vs 29.6%, P<0.05) were also significantly higher when TUDCA-treated cells were used as the nuclear donors. In addition, blastocysts derived from TUDCA-treated donor cells showed more total cell number (107.5±2.2 vs 93.9±1.3, P<0.05) and lower apoptotic index (2.2±0.7 vs 7.6±0.8, P<0.05) than that from control group (Fig 4). Regarding transcript abundance, the expression levels of *Hdac1*, *Dnmt1* and *Bax* were significantly lower in blastocysts produced from TUDCA-treated donor cells compared with control group, though there was no difference in expression level of *Ire1*, *Bip*, *Chop* or *Bcl-2* between these two groups (Fig 5).

**Table 4:** Development of SCNT reconstructed embryos when control or TUDCA-treated cells were used as the nuclear donors.

Groups	No. reconstructed oocytes	No. fused (%)	No. cleavage (%)	No. blastocyst (%)	Blastocyst cell number	Apoptotic index
Control	270	186 (68.9)^a^	161 (86.6) ^a^	55 (29.6)^a^	93.9±1.3 ^a^	7.6±0.8 ^a^
TUDCA	274	235 (85.8)^b^	219 (93.2) ^b^	89 (37.9)^b^	107.5±2.2 ^b^	2.2±0.7 ^b^

a,b: different superscripts within same column indicate significant difference (P<0.05).

## Discussion

Endoplasmic reticulum (ER) stress is a common phenomenon in cells and embryos cultured *in vitro*. There are many factors might cause *in vitro* cultured cells under adverse environmental conditions, and then trigger ER stress, such as: accumulation of reactive oxygen species (ROS) and cellular metabolites in the culture medium [30, 33]; change of temperature, oxygen and osmotic pressure [34, 35]; and procedures of cell passaging [36]. When ER stress occurs, it often leads to protein misfolding or accumulation of unfolded proteins within the endoplasmic reticulum, causing unfolded protein response (UPR) and disturbance of intracellular homeostasis [37]. If ES stress is too severe or lasts too long, accumulated unfolded or misfolded proteins might disturb normal physiological activities, and ultimately cause cumulative cellular damage, apoptosis and degeneration [19]. Previous study confirmed that ER stress or UPR was closely related to the expression of several ER-stress response genes, including *Bip* and *Chop* [38]. More evidences indicated that *Bip*, *Chop*, *PERK*, *Ire1* and *ATF6* are important molecules in ER stress signaling pathways, and can be regarded as biomarks of ER stress as well [30, 39, 40]. In the present study, we found routine serum starvation significantly increased the expressions of *Ire1*, *Bip* and *Chop* in the nuclear donor cells, and resulted in higher expression of pro-apoptosis marker *Bax* with more apoptotic cells. Our results indicate that serum starvation is a notable stress for *in vitro* cultured cells, which can trigger obvious ER stress and cellular damage. Our data also showed that TUDCA could significantly decrease the expressions of *Ire1*, *Bip* and *Chop* in nuclear donor cells under serum starvation condition, indicating that TUDCA effectively alleviates the ER stress triggered by serum starvation. Based on our scope of the present study, we only evaluated several routine biomarks of ER stress (*Ire1*, *Bip* and *Chop*), further studies will be essential to elucidate the specific pathway(s) involved in these nuclear donor cells under serum starvation.

For most species, donor cells are fused with enucleated recipient oocytes during SCNT procedure. Many factors are known to influence electrical fusion rate, such as electric parameters, composition of fusion medium, and temperature. In addition, the quality of cellular membranes (both donor cells and recipient oocytes) is another critical factor that determines electrical fusion rate [7, 41]. In the present study, identical electrical fusion conditions were applied and enucleated recipient oocytes were also from same batch throughout electrical fusion experiments. We found fusion rate was significantly higher when TUDCA-treated cells were used as the nuclear donors than that in control group. Though exact mechanism of this effect is not clear, we speculate that TUDCA treatment might improve cell viability and quality by alleviating ER stress and apoptosis, and finally enhance physical status of cell membrane and fusion rate.

It is well known that epigenetic status of donor nucleus is a key factor influencing reprogramming efficiency and success rate of SCNT cloning [42, 43]. The nuclear reprogramming process involves various epigenetic modifications, such as DNA methylation and histone modifications. Previous studies showed that increased global histone acetylation level and/or deceased DNA methylation level of the donor nucleus by histone deacetylase (Hdac) inhibitors or DNA methyltransferase (Dnmt) inhibitors can significantly improve the developmental capacity of SCNT embryos [44, 45]. In the present study, TUDCA-treatment not only reduced the ER stress but also refined the epigenetic modifications of nuclear donor cells under serum starvation condition. After TUDCA treatment, the expression levels of *Dnmt1* and *Hdac1* were significantly decreased, and the global level of H3K9ac was significantly increased. In addition, treatment of nuclear donor cells with TUDCA significantly improved development of SCNT embryos in terms of cleavage rate, blastocyst formation rate, total blastomere number and apoptotic index in day 7 blastocysts. These results are consistent with previous reports that nuclear donor cells at status of low global DNA methylation and high histone acetylation support better reprogramming and developmental potency of SCNT reconstructed embryos [32, 46]. Regarding the changes of epigenetic modifications in nuclear donor cells during TUDCA treatment, the mechanism is not clear yet. Interestingly, it has been reported that ER stress could induce the increase of certain HDACs [47], and TUDCA could reverse some cellular anomalies as well as increased histone deacetylases caused by ER stress [48]. Also, it has been reported that histone deacetylase inhibitors and DNA methyltransferase inhibitors can regulate not only cellular reprogramming, but also many other processes and events in various types of cells cultured *in vitro* [49–52]. Accordingly, we speculate that improvement of epigenetic reprogramming triggered by TUDCA might be the beneficial effect of its anti-ER stress function.

In conclusion, our experiments treatment of nuclear donor cells with TUDCA can significantly relieve ER stress and improve epigenetic modifications of nuclear donor cells under serum starvation condition, as well as significantly enhance following development of SCNT reconstructed embryos derived from these donor cells in cattle.
